# Exercise-Stress Echocardiography Reveals Systolic Anterior Motion of the Mitral Valve as a Cause of Syncopes in a Cardiac Amyloidosis Patient

**DOI:** 10.1155/2016/3198715

**Published:** 2016-11-10

**Authors:** Tor Skibsted Clemmensen, Henning Mølgaard, Niels Frost Andersen, Steen Baerentzen, Steen Hvitfeldt Poulsen

**Affiliations:** ^1^Department of Cardiology, Aarhus University Hospital, Aarhus, Denmark; ^2^Department of Hematology, Aarhus University Hospital, Aarhus, Denmark; ^3^Department of Pathology, Aarhus University Hospital, Aarhus, Denmark

## Abstract

Patients with cardiac amyloidosis are at increased AV-block and syncope risk. Therefore, a prophylactic pacemaker is often implanted. However, this case illustrates that other mechanisms should be ruled out prior to pacemaker implantation. The patient studied had mitral valve thickening without increased left ventricular outflow track (LVOT) velocity. However, bicycle exercise-stress test with simultaneous echocardiography revealed a stepwise decrease in blood pressure, a substantial increase in the LVOT velocity, and severe systolic anterior motion of the mitral valve. The patients' symptoms were likely explained by these findings. Therefore, a comprehensive clinical evaluation is warranted prior to pacemaker implantation in cardiac amyloidosis patients.

## 1. Case

A 71-year-old previous healthy man was referred to cardiac evaluation due to symptoms of dyspnea, orthostatic dizziness, and episodes of postural related syncope during a period of 2 months.

**Figure 1:**
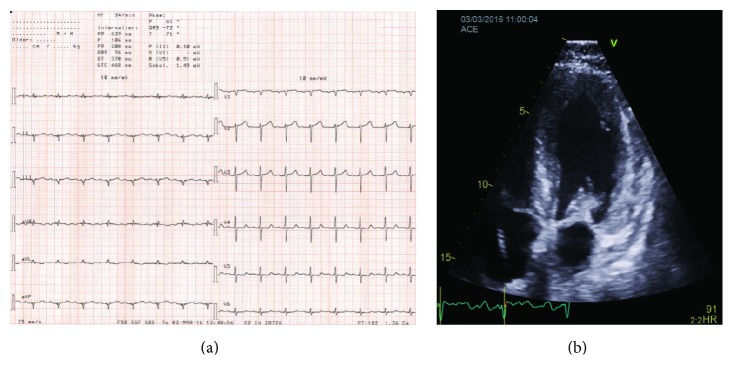
(a) Electrocardiogram. (b) Echocardiographic apical four-chamber view.

**Figure 2:**
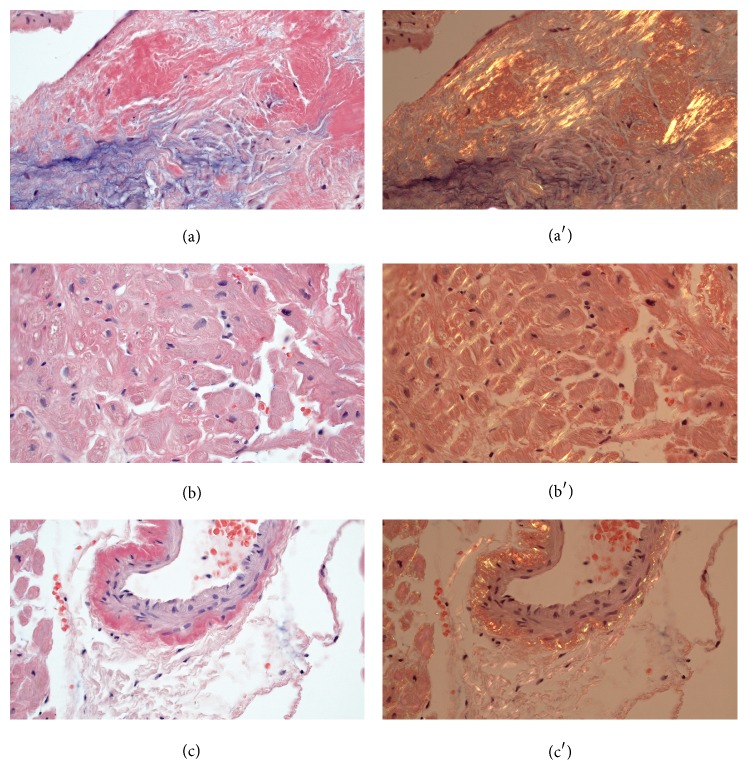
Endomyocardial biopsies. (a) Endocardial infiltration, congo stain; (a′) endocardial infiltration, congo stain and polarization; (b) interstitial amyloid deposits, congo stain; (b′) interstitial amyloid deposits, congo stain and polarization; (c) intramural coronary vessel, congo stain; (c′) intramural coronary vessel, congo stain and polarization.

Electrocardiogram revealed low-voltage limb leads and first degree of AV-block (Figure 1(a)). Echocardiogram revealed thickened left ventricle (LV) walls with a septal thickness of 18 mm and posterior wall of 16 mm (Figure 1(b)). The LV cavity was small with end-diastolic diameter of 34 mm and end-systolic diameter of 21 mm. The mitral valve was thickened and mild systolic anterior motion (SAM) was noted. However, left ventricular outflow track (LVOT) velocity was normal at 1.3 m/s both at rest and during the Valsalva maneuver. Ejection fraction was 70% but long-axis function severely reduced with global longitudinal strain at −10.1%. Apical sparing was noted. Coronary flow reserve was measured by Doppler echocardiography. CFR was severely reduced at 1.2 (normal range >2.5–4.5). Cardiac amyloidosis (CA) was suspected and the patient was referred for endomyocardial biopsy. This revealed extensive amyloid infiltration (Figure 2). Immunohistochemistry was positive for *λ*-light chains and negative for transthyretin, amyloid A, and *κ*-light chains.

**Figure 3:**
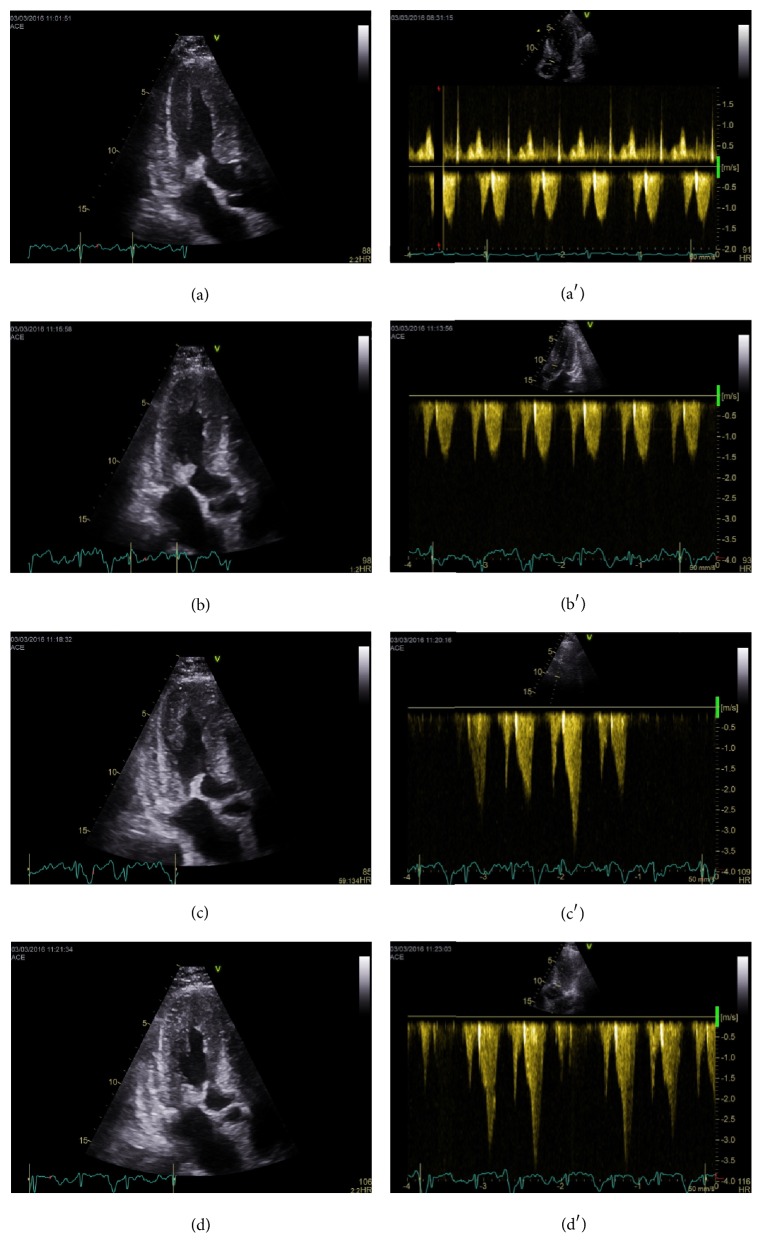
(a) Long-axis view at rest. (a′) Corresponding left ventricular outflow tract velocity. (b) Long-axis view at 10 Watts. (b′) Corresponding left ventricular outflow tract velocity. (c) Long-axis view at 30 Watts. (c′) Corresponding left ventricular outflow tract velocity. (d) Long-axis view at peak exercise. (d′) Corresponding left ventricular outflow tract velocity.

Pacemaker implantation was considered due to the previous syncopes and the findings of first degree of AV-block. However, we decided to evaluate the hemodynamics of the patient during a semisupine bicycle exercise-stress test with simultaneous echocardiography. At rest, blood pressure (BP) was 108/68 mmHg and heart rate (HR) was 90 beats/min. At 10 Watts BP was 98/61 mmHg, HR was 94 beats/min, and LVOT velocity was still normal at 1.5 m/s. At 30 Watts BP declined to 88/51 mmHg, HR was 98 beats/min, and LVOT velocity was between 2.0 and 3.5 m/s with a shark fin pattern. At peak exercise (40 Watts), BP was 74/45 mmHg and HR 106 beats/min. Severe SAM was noted at this point and the patients felt dizziness and dyspnea. The LVOT velocity was between 2.5 and 3.6 m/s (Figure 3).

The present case illustrates that mitral leaflet SAM should be suspected in CA patients with dizziness and syncopes, especially if echocardiography shows involvement of both the myocardium and mitral valve. This is important as CA patients with syncopes traditionally receive prophylactic pacemaker implantation. Previous case reports have demonstrated obstruction of the left ventricular outflow tract in CA patients [1–3]. The underlying mechanism of SAM in CA patients is unclear. It could involve a Venturi effect due to increased outlet tract flow mediated by increased septal thickness. This effect may be enlarged in CA patients due to the small LV cavities and increased heart rate. Furthermore, the amyloid deposit-induced increased leaflet thickness and papillary muscle displacement may also be involved. In the present case, symptoms and SAM were not present during resting conditions or during the Valsalva maneuver. However, we noted a stepwise drop in BP with the exercise induced SAM progression, which clearly illustrates the hemodynamic significance of the SAM. Therefore, exercise-stress echocardiography should be considered in the clinically evaluation of CA patients prior to prophylactic pacemaker implantation. Other causal symptom mechanisms such as conduction disturbances, ventricular arrhythmias, orthostatic hypotension, or vasovagal effects could be involved in the present case. However, Holter monitoring revealed no arrhythmias and no pacemaker was implanted in the patient.

Optimal treatment of left ventricular outflow tract obstruction in CA patients is difficult. The risk of surgical myectomy or mitral valve plasty is considered to be significantly increased [3]. The effect of alcohol septal ablation in CA patients is unknown. However, the patient was found unlikely to benefit alcohol septal ablation due to the severely reduced CFR, the pronounced SAM, and the small LV cavity. Hypertrophic obstructive cardiomyopathy patients often have near normal CFR [4] and slightly dilated LV cavity, which may be important for the effect of alcohol septal ablation. Disopyramide may reduce the left ventricular outflow tract obstruction in CA patients, but the negative inotropic effect may lead to further decrease of long-axis function [3]. We initiated treatment with Midodrine 5 mg × 3 and Metoprolol-Succinate 25 mg × 1. Furthermore, Dexamethasone and Melphalan therapy was initiated with satisfying hematological response. At three weeks of control the patient described no further syncopes. However, he suffered continuously from dizziness. The long-term Midodrine and Metoprolol-Succinate effect on SAM in CA patients is unknown.

In conclusion, mitral leaflet SAM should be ruled out prior to pacemaker implantation in CA patients. Furthermore, exercise-stress echocardiography may prove benefit in the clinical surveillance of CA patients.
